# Prognostic factors and overall survival among patients with ovarian cancer in the pre-PARP inhibitor era: the OCRWE-Finland study

**DOI:** 10.2340/1651-226X.2024.40324

**Published:** 2024-10-16

**Authors:** Mari Lahelma, Heini Rauhamaa, Riikka-Leena Leskelä, Outi Isomeri, Juhana Idänpään-Heikkilä, Sari Käkelä, Nichola Roebuck, Barbara Mascialino, Sakari Hietanen, Mikko Loukovaara, Annika Auranen

**Affiliations:** a Nordic Healthcare Group, Helsinki, Finland; b GSK, Helsinki, Finland; c GSK, Brentford, Middlesex, UK; d GSK, Verona, Italy; e Department of Gynecologic Oncology, Turku University Hospital and FICAN West, Turku, Finland; f Department of Obstetrics and Gynecology and Comprehensive Cancer Center, Helsinki University Hospital and University of Helsinki, Helsinki, Finland; g Department of Obstetrics and Gynecology, Tays Cancer Centre, Tampere University Hospital and Tampere University, Tampere, Finland

**Keywords:** Ovarian cancer, high-grade serous ovarian cancer, real-world evidence, overall survival, neoadjuvant chemotherapy, bevacizumab, prognostic factors

## Abstract

**Background:**

Despite recent treatment advances in ovarian cancer (OC), more real-world evidence studies investigating patient outcomes are needed. OCRWE-Finland was an observational cohort study investigating OC outcomes in Finland during the pre-PARP inhibitor era.

**Patients:**

Patients were diagnosed with OC between 2014 and 2019 in Finland. This analysis reports baseline characteristics of all patients, patients with high-grade serous OC (HGSOC), and overall survival (OS) for patients with HGSOC.

**Results:**

Among 1,711 patients diagnosed with OC, 867 (51%) had HGSOC. The absence versus presence of visible residual disease post-debulking surgery was associated with improved OS for patients at stage III (*n* = 303; median: NR *vs.* 43 months; *p* = 0.005), but not stage IV (*n* = 118; median: 37 months *vs.* 40 months; *p* = 0.96). Bevacizumab treatment at any line at stages III/IV improved OS in the short-term only. Receiving versus not receiving bevacizumab at first-line for patients with visible residual disease post-debulking surgery was associated with improved OS at stage III (median: 48 months *vs.* 36 months; *p* = 0.003), but not stage IV (median: 42 months *vs.* 37 months; *p* = 0.26). Multivariate Cox regression analyses showed that stage IV at initial diagnosis and the presence of R2 classification post-debulking surgery resulted in poorer OS.

**Interpretation:**

In the pre-PARP inhibitor era, the absence versus presence of visible residual disease post-debulking surgery was associated with improved OS in stage III, but not stage IV HGSOC. First-line bevacizumab seemed to be beneficial in patients with stage III HGSOC and visible residual disease.

## Introduction

Ovarian cancer (OC) is the second-leading cause of gynaecological cancer mortality worldwide [1]. Patients with OC often present with unspecific gastrointestinal-related symptoms, resulting in late diagnosis at more advanced stages [2, 3]. In Finland, between 2017 and 2021, approximately 590 women per year were diagnosed with OC and 383 women per year died because of OC [4]. Over this period, the 1- and 5-year survival rates were estimated at 83% and 47%, respectively [4].

Factors associated with improved overall survival (OS) in patients with epithelial OC include serous or endometrioid histology, lower stage at diagnosis, decreased volume of visible residual disease after surgery, favourable Gynaecologic Oncology Group performance status, mutated breast cancer genes 1/2 (*BRCA*mut), and younger age [5–7].

The first-line management of advanced OC (stage III/IV) involves cytoreductive surgery and neoadjuvant or adjuvant chemotherapy, including platinum-based compounds (e.g. carboplatin) or platinum–taxane combinations (e.g. carboplatin and paclitaxel) [8, 9]. Despite optimal upfront surgery and first-line platinum-based chemotherapy, approximately 70% of women with OC, especially those with stage III/IV high-grade serous OC (HGSOC), will relapse within 3 years of diagnosis [10–13]. As disease relapse is very common, subsequent maintenance therapies eventually become imperative for most patients [14].

Targeted maintenance treatments currently approved in Europe and the United States for patients with recurrent OC include treatment with bevacizumab and the introduction of a poly (ADP-ribose) polymerase inhibitor (PARPi; niraparib, olaparib or rucaparib) immediately after the patient achieves a response to chemotherapy [14]. Bevacizumab is an anti-vascular endothelial growth factor (VEGF) monoclonal antibody that has demonstrated promising results in first-line therapy when added to standard chemotherapy and also when used in the relapsed setting for both platinum-sensitive and platinum-resistant relapsed disease [10]. In Finland, bevacizumab was approved in January 2005, in alignment with the European Medicines Agency therapeutic indications. Although bevacizumab has shown improved progression-free survival (PFS) in various large randomised clinical trials, OS data are currently limited to a retrospective sub-analysis of high-risk patients (ICON7 trial) and a randomised phase III trial (GOG-0218) [10, 15, 16]. In addition, the ICON7 and GOG-0218 trials showed some evidence that bevacizumab discontinuation was associated with patients experiencing a rebound effect characterised by increased disease progression [17]. The use of PARPis as maintenance treatment options in both first-line (since March 2020) and second-line (since December 2017) settings, following response to chemotherapy, has been gradually increasing in Finland.

Despite the recent positive outcomes from clinical trials examining the use of maintenance therapies in OC, few studies have evaluated disease progression and OS in real-world clinical practice [18–21]. Obtaining real-world evidence (RWE) data will be beneficial for patients with OC, healthcare professionals, and payers. The aims of the OCRWE-Finland study were to describe the baseline demographic and disease characteristics, time to next treatment (TTNT; used as proxy for PFS), healthcare resource utilisation (HCRU), OS, and association between key prognostic factors using RWE data from patients diagnosed with OC in Finland between 2014 and 2019. Specifically, this article reports findings related to OS, and prognostic factors associated with OS, in patients with stage I–IV HGSOC, as they are known to be at higher risk than other histological types due to having a very distinct disease biology. Outcomes relating to TTNT can be found in the corresponding article by Mari Lahelma et al. [22] and results on HCRU will be published subsequently elsewhere.

## Methods

### Overview of OCRWE-Finland study design

This was a multicentre, retrospective cohort study based on secondary use of healthcare data from hospital medical records in Finland. The study involved the population of patients with OC from the three largest University Hospitals in Finland (Helsinki University Hospital [HUS], Turku University Hospital [VSSHP] and Tampere University Hospital [PSHP]), which treat approximately 50% of all patients with OC in Finland. Treatment of OC is largely centralised to University Hospitals, reflecting the standard of care in Finland. The target sample size for this study was approximately 1,650–2,100 patients. This number was determined sufficient for key descriptive analyses to be representative of the sample population as a whole whilst considering the feasibility of patient inclusion. The study complied with the ethical principles of the Declaration of Helsinki and the requirements of the European Union General Data Protection Regulation. Patient informed consent was not required, as the study was conducted under the Act on the Secondary Use of Health and Social Data.

This study included adult females (≥ 18 years of age) who were diagnosed with OC (i.e. ovarian, fallopian tube, or primary peritoneal cancer) between 1 January 2014 and 31 December 2019, and whose home municipality was located near HUS, VSSHP, or PSHP. Potentially eligible patients were identified by diagnosis codes (International Classification of Diseases, Tenth Revision [ICD-10]) from hospital records/hospital databases at the participating centres. The site-specific diagnosis codes for patient inclusion were C48, C56, and C57.0 for HUS and VSSHP, and C56, C57.0, and C57.8 for PSHP. Further information on study assessments, data management and analysis, and minimisation of bias can be found in the Data Supplement.

### Analysis of OS

This publication reports the OS results relating to the secondary objective of OCRWE-Finland. As HGSOC was the most prevalent histology among patients in OCRWE-Finland, this publication focuses on the OS results for the HGSOC cohort. OS was defined as the time between the date of diagnosis and the date of death. Date of death (all causes) was captured as recorded in medical records; patients not recorded as having died and those lost to follow-up were censored at the end of the study period (31 December 2019). Further information on overall OCRWE-Finland study endpoints can be found in the Data Supplement.

### Statistical analysis

Descriptive statistical data are presented as mean (standard deviation [SD]) for continuous variables and *n* (%) for categorical variables. The statistical tests used for subgroup comparisons were unpaired Student’s t test for continuous variables and Chi-squared test for categorical variables. Kaplan–Meier survival analysis was used to estimate the probability of OS, the log-rank test was used to compare survival distributions between subgroups, and a Cox proportional hazards (PH) regression analysis was applied to identify prognostic factors for OS, through univariate and multivariate analyses. Each explanatory variable was first assessed through univariate analysis, and then, significant variables were introduced to the multivariate analysis to identify independent prognostic factors for OS. Prior to analysis, the PH assumption was tested for covariates based on scaled Schoenfels residuals and graphical diagnostics. Analysis was performed with Rstudio, R version 4.1.0. (R Core Team 2021, R Foundation for Statistical Computing) and a *p*-value < 0.05 was considered statistically significant.

## Results

### Patient characteristics

In the OCRWE-Finland study, a total of 1,711 patients with OC diagnosed between 2014 and 2019 were included in the analysis, and the average age ± SD was 65.9 ± 13.4 years. The most common primary tumour site at diagnosis was ovary (75%). Among 1,418 patients with epithelial OC, 61% (*n* = 867) had HGSOC. Full descriptions of patient demographics and clinical characteristics for the overall population and patients with HGSOC are detailed in Table 1. Henceforth, only data on patients with HGSOC will be presented.

**Table 1:** Patient demographics and clinical characteristics.

	All patients (*N* = 1,711)	Patients with HGSOC (*n* = 867)
**Demographic characteristics**		
Mean age at first diagnosis (SD), years	65.9 ± 13.4	68.6 ± 10.8
Stage		
I	381 (22.3)	92 (10.6)
II	89 (5.2)	45 (5.2)
III	575 (33.6)	442 (51.0)
IV	291 (17.0)	203 (23.4)
BMI, *n* (%)		
Underweight: < 18.5 kg/m^2^	38 (2.2)	18 (2.1)
Normal weight: 18.5–24.9 kg/m^2^	617 (36.1)	327 (37.7)
Overweight: 25–29.9 kg/m^2^	510 (29.8)	285 (32.9)
Obese: > 30.0 kg/m^2^	361 (21.0)	169 (19.5)
Unknown	185 (10.8)	68 (7.8)
Geographic region, *n* (%)	1,042 (60.9)	522 (60.2)
Helsinki	363 (21.2)	220 (25.4)
Tampere	306 (17.9)	125 (14.4)
Turku		
**Clinical characteristics**		
Location at initial diagnosis, *n* (%)		
Ovaries	1,281 (74.9)	586 (67.6)
Fallopian tubes	107 (6.3)	95 (11.0)
Adnexa, others	84 (4.9)	67 (7.7)
Peritoneum and retroperitoneum	239 (14.0)	119 (13.7)
Histological grading, *n* (%)		
Serous, high-grade	867 (50.7)	867 (100)
Serous	212 (12.4)	–
Serous, low-grade	58 (3.4)	–
Mucinous	107 (6.3)	–
Endometrioid	101 (5.9)	–
Clear cell	73 (4.3)	–
Mesenchyme	66 (3.9)	–
Other	227 (13.3)	–
*BRCA* mutation status, *n* (%)		
* BRCA*mut	30 (1.8)	28 (3.2)
* BRCA*wt	282 (16.5)	212 (24.5)
Unknown	1,399 (81.8)	627 (72.3)
Residual tumour^a^, *n* (%)		
R0	471 (39.5)	270 (31.1)
R1	174 (14.6)	141 (16.3)
R2	133 (11.2)	107 (12.3)
Unknown	413 (34.7)	349 (40.3)

a The residual tumour status is presented only for patients who underwent surgery.

BMI: body mass index; HGSOC: high-grade serous ovarian cancer; OC: ovarian cancer; SD: standard deviation.

In total, 867 (51%) patients had HGSOC and were included in the OS analysis. The mean age at diagnosis ± SD was 68.6 ± 10.8 years. More than 70% (*n* = 645) of patients had stage III or IV disease. In total, 52% of patients were overweight or obese and 38% of patients had normal weight at diagnosis. In the vast majority of patients, *BRCA* mutation status was unknown (72.3%) and was more likely to have been tested in patients diagnosed in more recent years. The proportion of patients with known *BRCA* status increased from 10% in 2014 to 44% in 2019. The proportions of patients with known *BRCA* status who had *BRCA*mut or *BRCA*wt disease were 3.2% (*n* = 28) and 24.5% (*n* = 212), respectively (Table 1).

### Treatment patterns

Detailed information on the treatment patterns in this patient population can be found in the Data Supplement and the corresponding TTNT manuscript by Mari Lahelma et al. [22].

### OS by tumour stage

**Figure 1:**
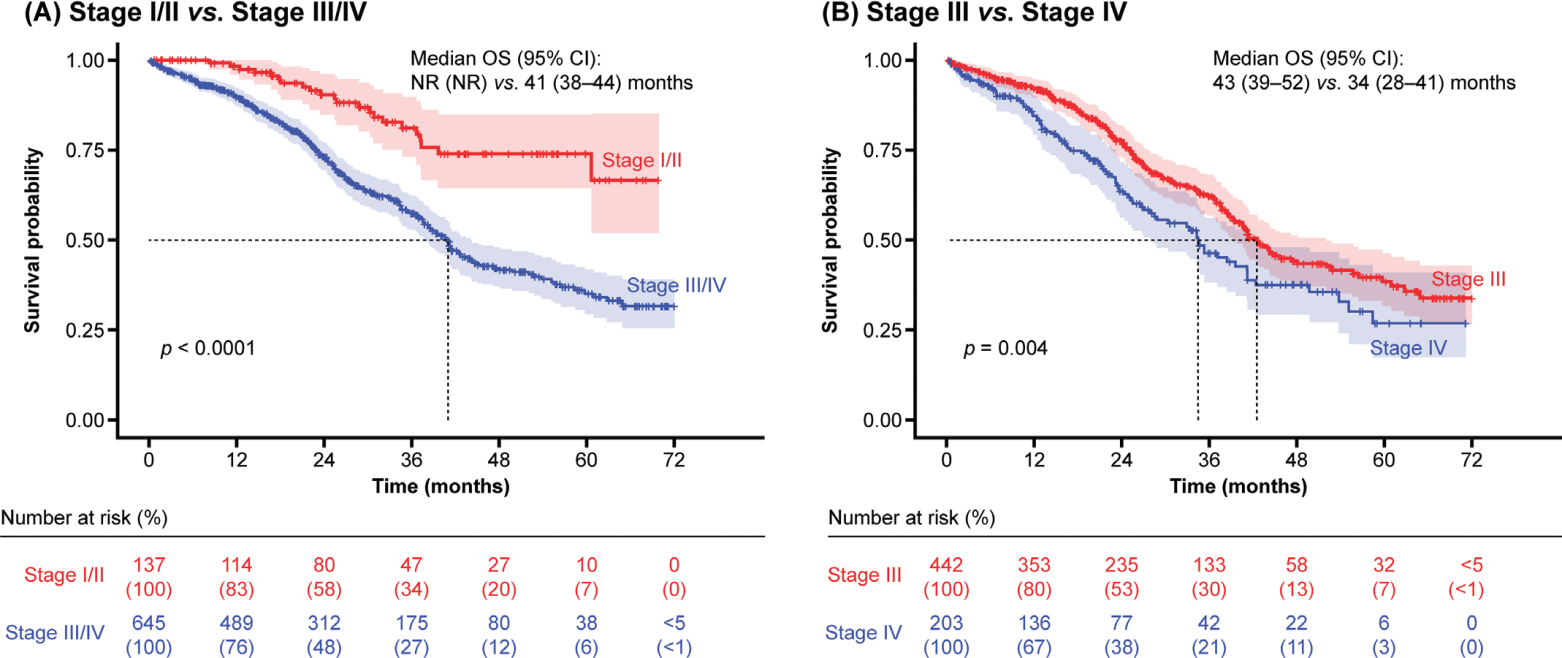
OS by stage of disease. CI: confidence interval; NR: not reached; OS: overall survival.

Patients with stage I/II disease had a significantly longer OS (median: not reached by 31 December 2019) compared with those who had stage III/IV disease (median: 41 months; *p* < 0.0001) (Figure 1A). In addition, OS was significantly longer in those with stage III disease compared with stage IV disease (median: 43 months *vs.* 34 months; *p* = 0.004) (Figure 1B).

### OS by tumour stage and visible residual disease status post-debulking surgery

**Figure 2:**
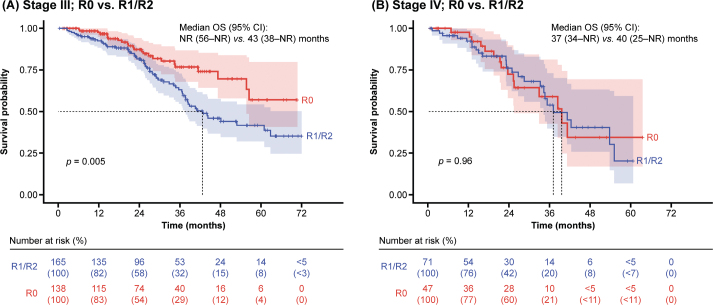
OS in patients at stage III and IV by presence or absence of visible residual disease status post-debulking surgery. CI: confidence interval; NR: not reached; OS: overall survival; R: residual tumour.

In patients with stage III disease, the absence of visible residual disease post-debulking surgery was associated with prolonged survival, as median OS was not reached after 6 years (by 31 December 2019), compared with OS when residual disease was present (R1 if < 1 cm and R2 if ≥ 1 cm after surgery) (median: 43 months; *p* = 0.005) (Figure 2A). However, in patients at stage IV, the absence of residual disease post-debulking surgery did not confer an OS benefit (median: 40 months *vs.* 37 months; *p* = 0.96) (Figure 2B).

### OS by tumour stage and prior treatment

**Figure 3:**
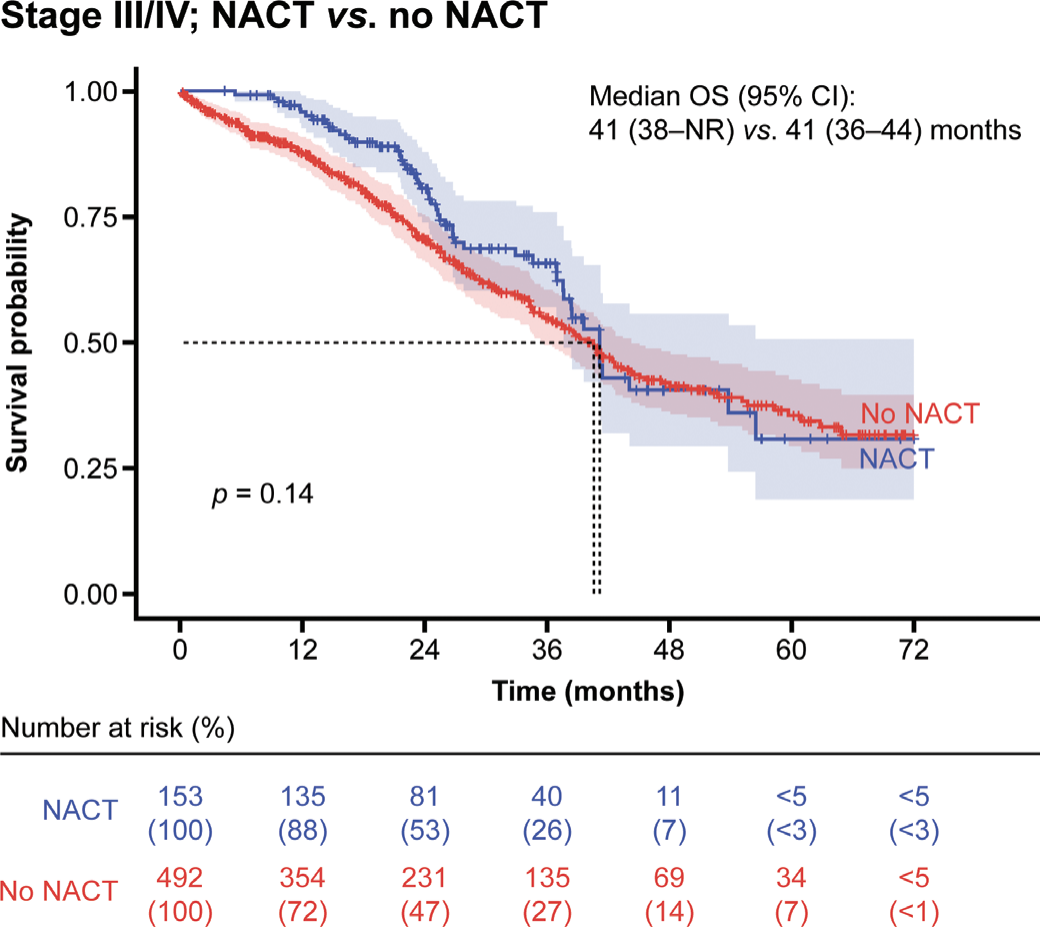
OS in patients at stage III/IV by use of NACT as first-line treatment. CI: confidence interval; NACT: neoadjuvant chemotherapy; NR: not reached; OS: overall survival.

The use of neoadjuvant chemotherapy (NACT) as first-line treatment in patients with stage III/IV disease did not result in an improvement in OS compared with those who did not receive NACT, although there was a trend towards improvement in the first 2 years (median: 41 months *vs.* 41 months; *p* = 0.14) (Figure 3).

**Figure 4:**
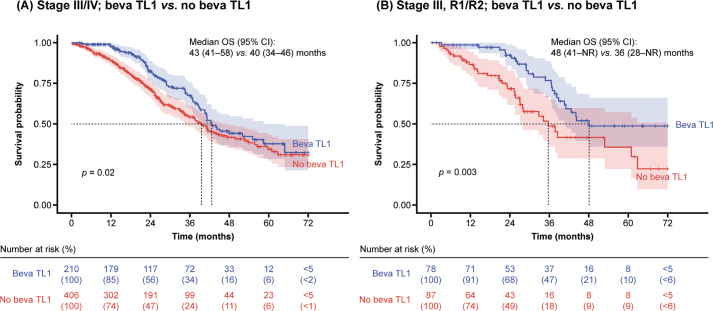
OS by use of first-line bevacizumab. Patients at stage III/IV disease receiving bevacizumab at TL1 showed improved OS during the first 3 years, but not after. This outcome may be attributed to a ‘rebound effect’ occurring when bevacizumab is discontinued. TL1: treatment line 1; Beva: bevacizumab; CI: confidence interval; NR: not reached; OS: overall survival; R: residual tumour.

Treatment with bevacizumab at any treatment line was associated with better OS in the first 3 years, but not thereafter, at stage III/IV disease (median: 43 months *vs.* 36 months; *p* = 0.0001) (Supplementary Figure S1). In addition, there was a statistical difference in OS between patients with stage III/IV disease who had and had not received first-line bevacizumab (median: 43 months *vs.* 40 months; *p* = 0.02) (Figure 4A). Patients receiving bevacizumab in the first line, compared with those who did not, were younger in age (mean age 65.3 years *vs.* 69.1 years, respectively; *p* < 0.001) and more often had advanced disease (93% *vs.* 70% had stage III/IV disease, respectively) and visible residual tumour (55% *vs.* 21%, respectively; *p* < 0.001). Treatment with first-line bevacizumab in patients with stage III disease and visible residual tumour (*n* = 78) resulted in improved OS compared with those who did not receive first-line bevacizumab (median: 48 months *vs.* 36 months; *p* = 0.003) (Figure 4B). Almost 60% of patients with stage IV disease and visible residual tumour received first-line bevacizumab, but this was not associated with significantly improved OS compared with those who did not receive first-line bevacizumab (median: 42 months *vs.* 37 months; *p* = 0.26).

### Prognostic factors for OS

The univariate analysis of prognostic factors showed that older age (hazard ratio [HR]: 1.03; 95% confidence interval [CI]: 1.02–1.04), stage III/IV disease at initial diagnosis, and the presence of visible residual tumour post-debulking surgery, were significantly associated with a poorer OS (Table 2). Patients with stage III disease had more than double and those with stage IV disease had more than three-times-greater risk of death compared with those with stage I disease. Body mass index (BMI), *BRCA* mutation status, use of NACT, and first-line bevacizumab were not significantly associated with OS in the univariate analysis. The multivariate analysis showed that stage IV disease at initial diagnosis and R2 classification of residual tumour were significantly associated with a poorer OS (Table 3).

**Table 2:** Univariate analysis for OS.

Characteristics		Patients	HR	95% CI	*p*-value^b^
Age (years)	Mean (SD)	68.6 (10.8)	1.03	1.02–1.04	**< 0.001**
Stage	I	92	–	–	–
	II	45	0.74	0.29–1.88	0.524
	III	442	2.38	1.42–3.97	**0.001**
	IV	203	3.49	2.05–5.95	**< 0.001**
Residual tumour^a^	R0	270	–	–	–
	R1	141	1.78	1.19–2.66	**0.005**
	R2	107	2.54	1.69–3.84	**< 0.001**
BMI	< 18.5	18	–	–	–
	> 39.9	27	1.93	0.68–5.47	0.218
	18.5–24.9	327	1.01	0.41–2.47	0.986
	25–29.9	285	1.30	0.53–3.20	0.561
	30–34.9	102	1.31	0.52–3.33	0.565
	35–39.9	40	1.71	0.64–4.55	0.286
*BRCA* mutation status	*BRCA*wt	212	–	–	–
	*BRCA*mut	28	0.94	0.36–2.44	0.902
	Unknown	32	1.20	0.56–2.54	0.639
NACT	No neoadjuvant therapy	704	–	–	–
	Neoadjuvant chemotherapy	163	0.81	0.60–1.08	0.157
Bevacizumab in TL1	No	584	–	–	–
	Yes	227	0.89	0.69–1.15	0.375

a The residual tumour status is presented only for patients who underwent surgery.

b The overall *p-*values for categorical variables are < 0.001, < 0.001, 0.010, and 0.879 for stage, residual tumour, BMI, and *BRCA* mutation status, respectively.

BMI: body mass index; *BRCA*: breast cancer gene; CI: confidence interval; HR: hazard ratio; mut: mutated; NACT: neoadjuvant chemotherapy; OS: overall survival; SD: standard deviation; TL1: treatment line 1; wt: wild-type.

P-value <0.05 was considered to be statistically significant.

**Table 3:** Multivariate analysis for OS.

Characteristics		Patients	HR	95% CI	*p*-value
Age	Mean (SD)	67.6 (10.1)	1.01	1.00–1.03	0.135
Stage	I	57	–	–	–
	II	30	0.77	0.21–2.86	0.698
	III	303	1.52	0.74–3.14	0.252
	IV	118	2.44	1.14–5.22	**0.022**
Residual tumour	R0	264	–	–	–
	R1	139	1.38	0.90–2.12	0.142
	R2	105	2.11	1.36–3.26	**0.001**

CI: confidence interval; HR: hazard ratio; OS: overall survival; R: residual tumour; SD: standard deviation; TL1: treatment line 1.

P-value <0.05 was considered to be statistically significant.

Finally, to investigate potential bias in OS data due to shorter follow-up times for patients who were diagnosed later in the study period, all analyses were repeated, including only patients diagnosed between 2014 and 2017. The results showed no evidence of bias compared with those presented in this article (data not shown).

## Discussion

Currently, there is a paucity of RWE studies on OC in Finland and in the Nordic population, as well as an unmet need to describe the real-world OS of these patients. The OCRWE-Finland study results address this knowledge gap by documenting the real-world OS and prognostic factors associated with OS, based on secondary use of healthcare data from hospital medical records, in the treatment era before PARPis, when maintenance treatment options were limited to bevacizumab. Moreover, this study provides benchmark OS findings in patients treated with bevacizumab that will help in the interpretation of data from ongoing studies assessing patients receiving PARPis in Finland.

One of the main findings of our study is that disease stage at initial diagnosis and the presence of visible residual tumour were independent prognostic factors associated with OS. Our findings indicate that patients of older age who were stage III/IV and had visible residual disease post-debulking surgery had a worsened prognosis, despite the use of NACT and established standard of care treatments. Moreover, OS maturity was still not reached at data read-out for patients diagnosed with stage I/II disease when treated with the standard of care. Conversely, in patients at stage III/IV, treatment with surgery ± NACT or ± bevacizumab has proven insufficient in transitioning the prognosis from a terminal to a chronic disease. Therefore, there remains a high unmet need for improved therapeutic options in this patient population. More RWE research is needed to investigate the effects of the recent introduction of PARPis for the treatment of OC. The ongoing re-run of this study will provide insight into whether the increasing use of PARPis as maintenance treatment for patients with OC in Finland is effectively addressing this unmet need. These findings will contribute to advancing precision medicine and optimising individualised treatments based on biomarkers and chemosensitivity.

This article also reports OS in patients with stage I–IV HGSOC where, as expected, patients diagnosed with advanced (stage III/IV) disease had significantly lower OS compared with patients at stage I/II. Among patients with advanced disease, the presence of visible residual disease post-debulking surgery in those at stage III had a negative impact on OS. Notably, for patients at stage III and with no visible residual disease post-debulking surgery, median OS was not reached after 6 years. Conversely, the presence or absence of visible residual disease post-debulking surgery did not significantly affect OS in patients at stage IV. Similar results were observed in the TTNT outcomes when comparing stage III/IV disease with the absence or presence of visible residual tumour [22]. These outcomes may help to provide valuable guidance in making treatment decisions for debulking surgery depending on the disease stage.

A French retrospective study investigated clinical outcomes in 208 patients with stage IV epithelial OC who either underwent no surgery, primary debulking surgery, or a combination of NACT and interval debulking surgery. The authors found that debulking surgery showed improved PFS and OS compared with non-operated patients with stage IV OC; however, the analysis cannot be directly compared with our study, as it included patients with epithelial OC rather than patients with HGSOC specifically [23]. Our findings align with a recent retrospective study that compared clinical outcomes in 247 patients with stage III/IV OC who underwent standard surgery versus more extensive surgical procedures (ultra-radical surgery). The study found that ultra-radical surgery improved PFS and OS in patients with stage III disease (median: NR *vs.* 36 months; *p* = 0.009), but did not reach statistical significance in stage IV OC (median: 39 months *vs.* 32 months; *p* = 0.691) [24].

In our study, the use of NACT in patients with advanced disease showed a trend towards improved prognosis in the first 2 years, although longer-term effects were not as clearly detectable. Platinum resistance may have contributed to the lack of prolonged OS benefit, as several retrospective studies have associated NACT with higher risk of platinum resistance [25, 26]. The authors acknowledge that interpretation of this analysis is limited by the dataset, which lacked data on platinum resistance, and anticipate that the ongoing re-run of the study will provide further insight into the effect of NACT on OS.

Patients at stage III/IV who received treatment with bevacizumab at any treatment line appeared to show an improved OS in the short-term but not in the long-term. This is consistent with previous clinical trial data for bevacizumab. The ICON7 and GOG-0218 phase III randomised international clinical trials investigated clinical outcomes in women with OC treated with bevacizumab in combination with standard platinum-based chemotherapy [15, 16]. The results of ICON7 reported that bevacizumab did not improve OS in the study population as a whole (median: 58.0 months *vs.* 58.6 months; *p* = 0.85); however, an OS benefit was recorded in patients with poor prognosis (median: 39.7 months *vs.* 30.2 months; *p* = 0.03) [15]. Similarly, the GOG-0218 trial reported that patients who received bevacizumab plus chemotherapy compared with chemotherapy alone did not have improved OS. However, an exploratory analysis suggested that bevacizumab in combination with or given after chemotherapy, may be beneficial for patients with stage IV disease [16]. Similar to our study, both GOG-0218 and ICON7 trials showed that treatment with bevacizumab demonstrated an initial 10-month relative survival benefit in high-risk patients with OC [16]. A recent real-world Belgian study investigated the evolution of treatment patterns and survival of 2,034 patients with epithelial stage IV OC diagnosed between 2004 and 2017. The study reported that, despite the improved survival associated with the increased proportion of patients receiving debulking surgery over time (HR, 0.88; 95% CI: 0.79–0.98), the introduction of bevacizumab did not contribute to improved survival (HR, 0.94; 95% CI: 0.85–1.03) [27]. This observation underlines the importance of real-world data following the publication of clinical trial results.

In the OCRWE-Finland study, patients with stage III/IV disease treated with first-line bevacizumab had improved OS, especially during the first 3 years. The fact that the improvement becomes less pronounced over time may be attributed to the possible ‘rebound effect’ occurring when bevacizumab is discontinued [17]. Moreover, it is important to note that patients that received first-line bevacizumab were younger in age, especially in high-risk groups. The decision to treat younger patients with bevacizumab may be attributed to several factors, including the tendency of clinicians to pursue more aggressive treatment options for younger patients to achieve maximum benefit, and the contraindications for the use of bevacizumab in older patients due to co-morbidities. However, we believe this dataset was not suitable to identify in what sense the disease may have been more aggressive for this group of patients. In patients with stage III disease, but not stage IV, and visible residual tumour, treatment versus no treatment with first-line bevacizumab resulted in statistically significant OS improvement. However, it is worth noting that patients with visible residual tumour who do not receive bevacizumab are typically those who have previously had bowel surgery because of extensive disease, contributing to worse prognosis. Moreover, despite the tendency to group stages III and IV together, it is notable that patients with stage III disease showed a statistically significant OS improvement, whereas those with stage IV did not.

Limitations of this study are consistent with those of retrospective studies, including possible bias from missing data in medical records and inconsistencies within and across physician assessments. However, a strength of our study was that it allowed the effective observation and collection of data on real-world clinical practices of this specific treatment era. The long follow-up time allows the appreciation of OS in its full potential for patients treated with the standard of care at that time. In fact, clinical prospective studies require many years to capture such information and to reach mature OS read-outs; our results may help to contextualise results from recent clinical trials. To address any potential abstraction errors, source data verification was used; aggregated results within the data sources and manuscript were cross-checked against the original raw data in the research environment. Furthermore, the overall population of this study reflects the three largest hospitals in Finland (approximately 50% of patients with OC in Finland) but may not be representative of smaller national hospitals. In Finland, OC surgeries are centralised to university hospitals, but patients continue to receive maintenance treatment and follow-up care at their regional hospitals. Therefore, our study may lack follow-up data on patients who underwent surgery, potentially affecting the composition of our study population. The investigation of the potential role of *BRCA* mutations as predictive biomarkers for OS was limited by the poor coverage of *BRCA* status among patients included in this study (patients with HGSOC; 27.7% [*n* = 240] with known *BRCA* status of whom 11.7% [*n* = 28] were *BRCA*mut). In addition, the observation that complete resection did not affect OS at stage IV disease needs further investigation. The ongoing re-run of this study will further elucidate OS effects across different subgroups of patients considering the type of surgery and tumour site. Considering the retrospective nature of the study design, this finding was expected; our study was conducted during the pre-PARPi era, when PARPis were not reimbursed nor routinely used in Finland. The use of PARPis has gradually increased in Finland since 2018 following European Medicines Agency approvals and subsequent national reimbursement.

Finally, to determine whether patients who were diagnosed late in the study period may have introduced bias in OS data due to the shorter follow-up time, all the analyses were repeated, including only patients who were diagnosed between 2014 and 2017. There was no evidence of bias in this repeat analysis in OS data compared with results presented in this article (data not shown), underscoring the robustness of the study design and its findings.

Considering the retrospective and observational nature of this study, the selection of OS as the primary endpoint, along with the provision of results for different sub-populations at regular timepoints, provides relevant information for both patients and healthcare professionals. It is important to highlight that interpreting the treatment effect on OS, especially in advanced disease, can be challenging, owing to potential confounding factors arising from multiple subsequent lines of therapy [28]. While prolonging survival is the primary goal of cancer treatments, reporting alternative endpoints is essential to capture the full value of cancer therapies [29]. To address this, outcomes related to TTNT (as a proxy for PFS) are reported in the corresponding article by Mari Lahelma et al. [22]. Finally, the upcoming manuscript by Mari Lahelma et al. on HCRU will shed light on reimbursement decision-making. As the use of PARPis continues to increase in Finland, forthcoming real-world studies will report their impact on OS in patients with HGSOC.

## Conclusion

This is the first real-world study to comprehensively assess OS in relation to tumour stage, prior treatment, and visible residual tumour classification post-debulking surgery in patients with HGSOC in Finland in the pre-PARPi era. Furthermore, the insights from this study establish a valuable reference point for exploring the real-world impact of PARPis in this disease. Our study revealed that maintenance treatment with bevacizumab, when this was the only available maintenance treatment option, could improve OS in patients with visible residual disease post-debulking surgery at stage III but not stage IV. This finding may support physicians when making clinical decisions for patients with advanced disease.

## Supplementary Material

Prognostic factors and overall survival among patients with ovarian cancer in the pre-PARP inhibitor era: the OCRWE-Finland study

## Data Availability

Information on GSK’s data-sharing commitments and requesting access to anonymised individual participant data and associated documents from GSK-sponsored studies can be found at www.clinicalstudydatarequest.com.
