# Supplementary material for: Prognostic factors and overall survival among patients with ovarian cancer in the pre-PARP inhibitor era: the OCRWE-Finland study
Source: Acta Oncol. 2024 Oct 16;63:40324. doi: 10.2340/1651-226X.2024.40324 (PMC11495122; doi:10.2340/1651-226X.2024.40324)
Supplement: Prognostic factors and overall survival among patients with ovarian cancer in the pre-PARP inhibitor era: the OCRWE-Finland study [file AO-63-40324-s1.pdf]

Supplementary material has been published as submitted. It has not been copyedited, or typeset by Acta Oncologica.

Data Supplement

Supplementary methods

Overall OCRWE-Finland study endpoints

The primary objective of the study was to describe the time to next treatment (TTNT), used as proxy for (PFS) for patients with ovarian cancer (OC). Secondary endpoints of the study included characterisation of baseline patient demographics and disease characteristics, treatment pathways, distribution between OC subtypes, OC-related healthcare resource use, and describing disease progression and survival (measured by overall survival [OS]) and response to treatment (measured by complete response, partial response, and residual disease after surgery). The exploratory objective of the study involved quality assessment of the real-word dataset collected to support statistical testing in identifying prognostic factors associated with patient characteristics, prior treatments, OS, and TTNT.

Study assessments

Patient demographics and characteristics assessed in the study included age, body mass index (BMI), anatomical location of tumour at initial diagnosis (ovaries, fallopian tube, or primary peritoneal), histological classification (serous, low-grade serous, high-grade serous, mucinous, endometrioid, clear cell, mesenchyme, or other, including germ cell), disease stage, *BRCA* mutation status, and visible residual tumour after surgery. Visible residual tumours were categorised as R1 if < 1 cm and R2 if ≥ 1cm after surgery. Initial OC stage was determined based on the

International Federation of Obstetrics and Gynaecology (FIGO) staging classification.

For treatment pattern assessment, patients were grouped into categories for each line of treatment. One chemotherapy treatment was defined by combining all doses administered within a 59-day interval. The determination of the first and subsequent lines of treatment was based on combined data for surgery and chemotherapy. In the first-line, patients received either surgery alone, surgery plus adjuvant chemotherapy, neoadjuvant chemotherapy (NACT) plus surgery plus adjuvant chemotherapy, chemotherapy only, or other. In the second- and third-lines, patients received platinum-based chemotherapy and other chemotherapy. Maintenance therapies and anti-angiogenic treatments were not considered as separate lines of treatment.

The use of bevacizumab at each treatment line was defined based on whether administration of bevacizumab according to electronic health records was identified between the first and last date of each treatment line. One treatment course of bevacizumab was defined by combining all drug doses administered within a 59-day interval.

Data management and analysis

Data were extracted from patients' medical records and hospital databases within the study sites. Patient data were collected, combined, and provided in pseudonymised form by the Finnish Social and Health Data Permit Authority (Findata), the permit holder of the study (permission date: 17 February 2021). Findata operates under the Secondary Data Act, disclosing information in such a way that the data protection of the individual is maximised. Patients were

identified by a unique study code to preserve confidentiality. Standard imputation was applied for missing patient data from missing days and/or months (when the day was missing, the 15th of the month was assumed; when both day and month were missing, the 1st of July was assumed). For other missing data, only available data were analysed and no imputation was performed. The index-event date was the date of diagnosis. The median overall follow-up time (as defined as time from index date to death/censoring) was 22.4 months. The total overall follow-up time for the study population was 1821 years.

Minimisation of bias

Selection bias was minimised by analysis of the total population in the three University Hospitals participating in this study. Recall bias and transfer bias were addressed by using secondary data from reliable hospital data lakes and patients' medical records. Confounding bias was avoided by stratifying the study population and citations bias was minimised by employing a multidisciplinary research team in the study.

Supplement Figure S1. OS in patients at stage III/IV by prior use of bevacizumab in any treatment line

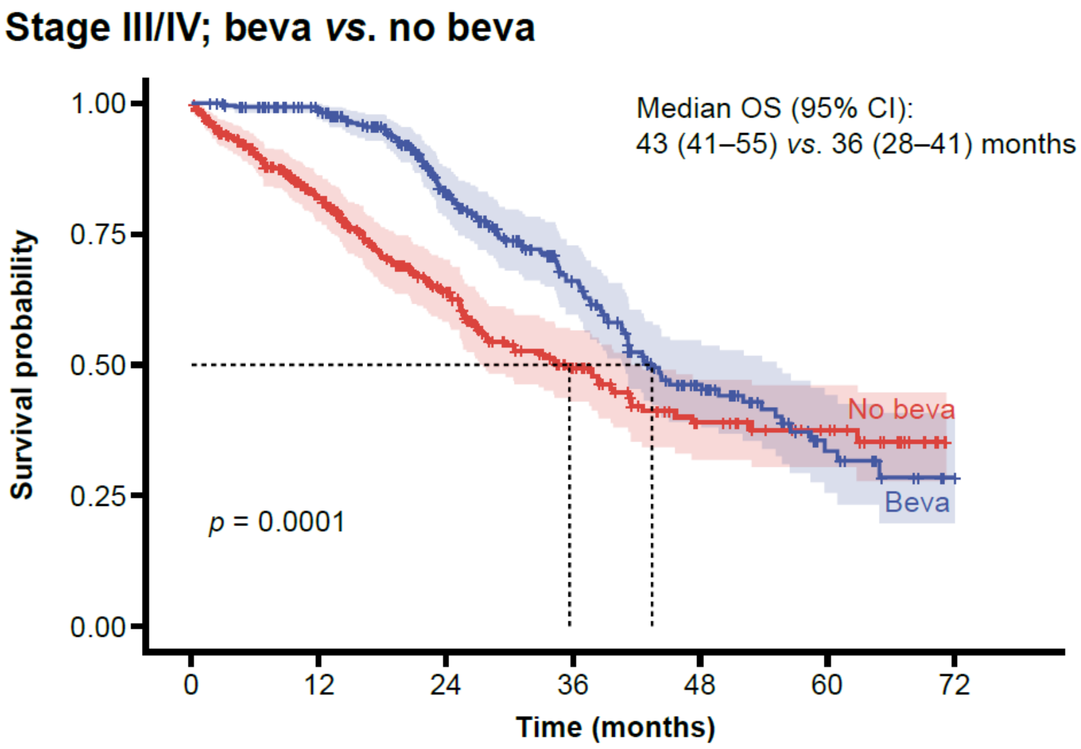

Number at risk (%)

Beva	296 (100)	264 (89)	179 (60)	105 (35)	47 (16)	17 (6)	<5 (<2)
No beva	349 (100)	225 (64)	133 (38)	70 (20)	33 (9)	21 (6)	0 (0)

Beva: bevacizumab; CI: confidence interval; OS: overall survival.
